# Psychometric evaluation of the Persian version of the quality of life in epilepsy inventory-31

**Published:** 2013

**Authors:** Navid Mohammadi, Shiva Kian, Farnoush Davoudi, Seyed Mohammad Ali Akbarian Nia, Marzieh Nojomi

**Affiliations:** 1 Associate Professor, Department of Community Medicine, Tehran University of Medical Sciences, Tehran, Iran; 2 Community and Preventive Medicine Specialist, Iran University of Medical Sciences, Tehran, Iran; 3 Assistant Professor, Department of Community Medicine, Tehran University of Medical Sciences, Tehran, Iran; 4 Assistant Professor, Department of Neurology, Iran University of Medical Sciences, Tehran, Iran; 5 Professor, Department of Community Medicine, Iran University of Medical Sciences, Tehran, Iran

**Keywords:** Quality of Life, Epilepsy, Quality of Life in Epilepsy

## Abstract

**Background:**

Health assessment in patients with epilepsy (PWE) should include both clinical outcomes and health related quality of life (HRQOL) measures. The quality of life (QoL) in epilepsy-31 inventory (QOLIE-31) is widely used for QOL studies in epilepsy. This study aims to evaluate psychometrics of the Persian version of the inventory (QOLIE-31-P).

**Methods:**

Following a standard forward-backward translation and cultural adaptation, the construct validity of the QOLIE-31-P was assessed by explanatory factor analysis, multi-trait scaling analysis, and known group comparison. The criterion validity was assessed by calculating the Pearson correlation to SF-36 (36-item short-form health survey). The reliability was assessed by calculating Cronbach's alpha and test-retest study.

**Results:**

The factor analysis extracted from 8 factors explaining 70.35% of the variations. Item-scale correlations revealed that individual items significantly had the strongest association with the domain they were loaded on. The Pearson coefficient of correlation between QOLIE-31-P and the overall scores of SF-36 was 0.876 (P < 0.0001). Patient with medically controlled seizures scored higher than those who experienced seizures during the previous year to study date (P < 0.0001). The Cronbach's *α* of overall QOLIE-31-P inventory was 0.9. The overall test-retest coefficient of correlation was 0.68 (P = 0.003).

**Conclusion:**

QOLIE-31-P is a valid and reliable tool to be applied in health assessment of patients with epilepsy.

## Introduction

Epilepsy is a chronic neurological disorder defined by frequent seizures, affecting more than 1,300,000 Iranians.^1^ The disease has significant adverse effects on patients’ physical, psychological and social functioning, even when seizures are medically controlled. Therefore, health assessment in patients with epilepsy (PWE) should include both clinical outcomes (type, severity and frequency of seizures, medication side effects) and health related quality of life (HRQOL) measures.

The quality of life in epilepsy-31 inventory (QOLIE-31) is a well known brief scale, driven from the original QOLIE-89 by RAND (*Research ANd Development*) Corporation, USA, designed to assess disease-specific and some general HRQOL issues in adults (> 18 years) PWE. The instrument and its cross-cultural translations have been and are currently used in many clinical and research settings. Regarding the three criteria used by Leone et al (i.e. validation, diffusion of use and specificity of domains) the inventory is amongst six preferred tools for quality if life (QOL) studies in epilepsy.^2^

In order to adapt the inventory to be widely used in Iranian adult PWE, the Persian version of QOLIE-31 (QOLIE-31-P) was developed and its psychometric characteristics were investigated in the present study.

## Materials and Methods

### Instruments

The QOLIE-31 is a questionnaire with 31 items, clustered in seven multi-item scales (i.e. overall quality of life, emotional well-being, energy/fatigue, cognitive functioning, medication effects, seizure worry, and social functioning) and one single item on overall health. The QOLIE-31 overall score was obtained using a weighted average of the multi-item scale scores. The scale scores were calculated according to scoring manual which also provided a scoring system for each item. The scale scores and overall score values range from 0 to 100, where higher values reflect more favorable states.^3^

The 36-item short-form health survey (SF-36) is a generic health survey tool for QoL which yields an 8-domain profile including bodily pain, general health, mental health, physical functioning, and role limitation due to emotional problems, role limitation due to physical problems, social functioning and energy/fatigue. The RAND SF-36 scoring tables are used for recoding items and averaging them to form scales which range from 0 to 100, with 100 representing the highest level of functioning possible.^4^

### Cross-cultural translation

A standard forward-backward translation method was used to develop the QOLIE-31-P. An expert panel of neurologists, community medicine specialist and bilingual English-Persian translators reviewed the draft for resolving the wording differences between the Persian and English versions and ensuring the content and face validity of the Persian version of the inventory. Prior to final administration, the QOLIE-31-P was tested in a preliminary study with 12 adult PWEs for simplicity, comprehensibility and acceptability.

### Study population

The study sample was recruited from adult PWEs (> 18 years) who attended Iranian Epilepsy Association (IEA). Patients were considered eligible if they had a proven diagnosis of primary generalized or partial epilepsy, were on antiepileptic medications, willing to participate, able to comprehend and complete the questionnaire.

### Analysis

The construct validity of the QOLIE-31-P was assessed by explanatory factor analysis (Principal Component Analysis as extraction of rotation method; Varimax with Kaiser normalization), multi-trait scaling analysis, and comparison of groups with different seizure frequencies during the previous year to study date (known group comparison).The criterion validity of the instrument was assessed by calculating the Pearson correlation to SF-36.

The reliability of the Persian version was assessed by calculating Cronbach's alpha for internal consistency and a test-retest (2-5 weeks later); besides the study was performed on a subgroup of patients.

### Administration

During the 6 months recruitment of the study, a trained nurse assessed visitors to IEA for eligibility, explained the study objectives, received the verbal consents and provided instructions for completing the questionnaires. Both QOLIE-31 and SF-36 questionnaires were completed by study participants in one session. A randomly selected group of 24 patients completed the QOLIE-31 once again, in a visit 2-5 weeks after they first completed the questionnaires, given that they experienced no significant changes in their general health condition.

## Results

### Descriptive statistics

The study sample comprised 84 PWEs, aged 18-60 years (mean ± SD = 32.6 ± 9.2), of whom 47 (56%) were females and 22 (26%) were married. 59% and 16.7% of the patients suffered from tonic-clonic and partial seizures, respectively. Participants were diagnosed with epilepsy for an average of 19.1 ± 16.9 years (1-43 years) and experienced 0-180 seizure episodes in the previous year to study date (Table 1).

**Table 1:** Demographic characteristics of the study subjects

Sex (%)	Male	37 (44)
	Female	47 (56)
Age; mean (SD)	Male	29.9 (9.2)
	Female	34.6 (8.9)
	Total	32.6 (9.2)
Marital status (%)	Single	61 (72)
	Married	22 (26.9)
	Divorced	1 (1.1)
Education (%)	< 12 years	32 (37.9)
	High school graduation	36 (41.8)
	University education	16 (20.3)
Occupation (%)	House wife (females only)	29 (61)
	Employee	19 (22)
	Self-employed	11 (13)
	Student	8 (9)
	Jobless	17 (20)
Type of epilepsy (%)	Tonic-clonic	49 (59)
	Partial	14 (16.7)
	Absence	11 (13.1)
	Others	10 (11.9)
Duration; mean (SD)	Male	16.1 (5.35)
	Female	22.1 (5.95)
	Total	19.1 (8.45)
Number of seizure attacks in the previous year (%)	Male	49 (27)
	Female	56 (31)
	Total	53.4 (29.5)

Note: SD = standard deviation; (%) = number (percentage)

The mean (± 2 SD) QOLIE-31 overall score of all responders was 52.2 (14.6). Male participants scored 52.2 (14.7) and females scored 52 (14.6). Mean scale scores ranged from 20 (energy/fatigue) to 59.84 (social functioning). The cognitive functioning had the strongest association with the overall score (r = 0.805, P < 0.0001) followed by social functioning (r = 0.797, P < 0.0001). Table 2 summarizes descriptive statistics of QOLIE-31 and Pearson correlation of sub-scales with overall scores.

**Table 2:** Descriptive statistics in quality of life in epilepsy-31 inventory and Pearson correlation of sub-scales with overall score

Scale	Mean (95% Confidence Interval)	Median	Pearson correlation with overall score (P)
Seizure worry	54.5 (28-81)	56	0.685 (< 0.0001)
Overall quality of life	36 (9.5-62.5)	40	0.521 (< 0.0001)
Emotional well-being	58.7 (39.2-87.2)	64	0.703 (< 0.0001)
Energy/fatigue	20 (11.25-28.75)	55	0.700 (< 0.0001)
Cognitive functioning	50.7 (32.8-68.6)	49.5	0.805 (< 0.0001)
Medication effects	42.28 (13.86-70.7)	36.1	0.562 (< 0.0001)
Social functioning	59.84 (37.42-82.26)	60	0.797 (< 0.0001)
Overall score	52.5 (38-67)	51.9	

Comparison of QOLIE-31 overall score made between groups regarding their sex, education, occupation and type of seizure revealed no statistically significant difference.

### Validity

The factor analysis on 30 items led to extraction of 8 factors with eigenvalues > 1, explaining 70.35% of the variations. The first factor was a heterogeneous one, containing loadings from “seizure worry”, “cognitive functioning”, “social function”, “medication effects” and “overall health” domains. The second factor included items from “seizure worry”, “energy/fatigue”, “cognitive functioning”, “social function” and “overall quality of life” domains. The third factor corresponded to “seizure worry” domain (4 out of 5 items of the domain). Factor 4 contained two “cognitive” and one “social functioning” factor. One item from each of energy/fatigue and emotional well-being domains was loaded on factor 5. Factor 6 consisted of two items from cognitive functioning. Factor 7 contained one item from each of overall QoL, energy/fatigue and emotional well-being domains. Factor 8 had just one item from medication effects. Item 20 (driving) was not included in any of the factors.

Item-scale correlations for seizure worry (r = 0.65-0.83), overall QoL (r = 0.59-0.95), emotional well-being (r = 0.58-0.81), energy/fatigue (r = 0.64-0.80), cognitive functioning (r = 0.46-0.76), medication effects (r = 0.63-0.84), and social function (r = 0.51-0.82) revealed that individual items significantly had the strongest association with the domain they were loaded on.

The Pearson coefficient of correlation between the QOLIE-31-P and SF-36 overall scores was 0.876 (P < 0.0001). The seizure worry scale of QOLIE-31-P demonstrated the strongest association with pain scale of SF-36 (r=.889, P < 0.0001). Table 3 summarizes the correlation of the two questionnaires’ subscales.

**Table 3:** Pearson correlation of quality of life in epilepsy inventory and SF-36 overall and subscale scores

		Social functioning	Role limitations due to physical problems	Role limitations due to emotional problems	Energy/fatigue	Emotional well-being	Physical functioning	Bodily pain	General health	SF36 overall score
Seizure worry	r*	0.143	0.363	0.437	0.718	0.281	0.262	0.889	0.331	0.672
	P	0.206	0.001	0.000	0.000	0.010	0.018	0.000	0.002	0.000
Overall quality of life	r*	0.223	0.677	0.282	0.096	0.381	0.251	0.004	0.443	0.430
	P	0.047	0.001	0.010	0.398	0.001	0.023	0.970	0.000	0.001
Emotional well-being	r*	0.244	0.627	0.283	0.280	0.657	0.486	0.271	0.581	0.645
	P	0.028	0.001	0.010	0.012	0.001	0.001	0.013	0.001	0.001
Energy/fatigue	r*	0.121	0.587	0.292	0.324	0.605	0.465	0.283	0.583	0.594
	P	0.282	0.001	0.007	0.004	0.001	0.001	0.010	0.001	0.001
Cognitive	r*	0.131	0.632	0.702	0.645	0.311	0.185	0.545	0.505	0.701
	P	0.246	0.001	0.001	0.001	0.004	0.096	0.001	0.001	0.001
Medication effects	r*	0.184	0.296	0.254	0.770	0.287	0.231	0.289	0.317	0.511
	P	0.105	0.007	0.021	0.001	0.009	0.038	0.008	0.004	0.001
Social Function	r*	0.243	0.529	0.615	0.796	0.360	0.379	0.459	0.472	0.718
	P	0.030	0.001	0.001	0.001	0.001	0.001	0.001	0.001	0.001
QOLIE-36 overall score	r*	0.274	0.785	0.660	0.733	0.568	0.448	0.587	0.664	0.876
	P	0.014	0.001	0.001	0.001	0.001	0.001	0.001	0.001	0.001

* Pearson correlation coefficient

Patient with medically controlled seizures scored higher than those who experienced seizures during the previous year to study date, respecting the overall scale scores, overall QoL, cognitive functioning, medication effects and social functioning sub scales (P < 0.0001).

### Reliability

The Cronbach's alpha of the overall QOLIE-31-P inventory was 0.9. The highest internal consistency was seen in seizure worry scale (α = 0.80), followed by energy/fatigue scale (α = 0.75).

The overall test-retest coefficient of the correlation was 0.68 (P = 0.003). The strongest subscales’ test-retest correlation was between medication effects (r = 0.728, P < 0.0001) followed by seizure worry domain (r = 0.711, P < 0.0001). Table 4 summarizes the reliability measures of the QOLIE-31 Persian version.

**Table 4:** Reliability measures of QOLIE-36

	Cronbach's alpha	Test re test Correlation (P)
Seizure worry	0.803	0.711 (< 0.001)
Overall quality of life	0.500	0.501 (0.329)
Emotional well-being	0.752	0.572 (0.020)
Energy/fatigue.	0.755	0.678 (< 0.001)
Cognitive	0.740	0.543 (0.006)
Medication effects	0.666	0.728 (< 0.001)
Social function	0.656	0.500 (0.016)
Overall	0.900	0.680 (0.003)

## Discussion

The factorial structure of the QOLIE-31 Persian version (8 factors) had remarkable similarities to other versions, specially the Serbian language one (6 factors). The two questionnaires had 4 identical factors and 2 others with minor differences.^5^

Regarding the multitrait scaling analysis results, the QOLIE-31-P had satisfactory construct validity, since all the items had stronger association with scales which they construct compared to other scales. Correlations with SF-36 indicated that the Persian adaptation of the inventory well reflected the general aspects of the HRQOL.

The QOLIE-31-P is a reliable instrument to measure HRQOL in PWEs as indicated by high Cronbach's alpha coefficient for the overall score. The coefficient was lower than desirable standard in medication effects, social function and overall QoL scales. The overall QoL scale contained only two items, which made it difficult to achieve adequate internal consistency. The social functioning scale contained items which were applicable only to a limited number of responders (i.e. those with a regular job or driving license) which in turn led to several missing values. Considerable variations in type and dosage of medications taken to control seizure attacks leads to variation of answers to medication effects items and low internal consistency of the scale.

The results of the reliability testing using Cronbach's alpha, were consistent with those reported in the American, German, Spanish, French, Czech, Serbian, Croatian, Greek, Thai, Chinese, Italian and Brazilian studies.^3, 5–16^

The test-retest analysis of the German and Italian versions revealed higher correlation coefficients compared to our results. The most probable explanation was remarkable number of unanswered left items in either “test” or “retest” rounds of administration.^7, 13^

There has been another study from Iran which reported the range of Cronbach's alpha from 0.71-0.89, except the medical effects and social functioning scales which demonstrated less desirable reliability. According to the mentioned study, the QOLIE-31 Persian version was able to discriminate groups of patients according to the type of seizures and frequency of episodes in the previous month and year.^17^

## Conclusion

The study findings indicated that the QOLIE-31 Persian version had favorable construct and criterion validity, high internal consistency and acceptable temporal stability, which made the inventory an appropriate instrument for health assessment in patients with epilepsy.
